# The Piwil1 N domain is required for germ cell survival in Atlantic salmon

**DOI:** 10.3389/fcell.2022.977779

**Published:** 2022-09-19

**Authors:** Almeida F. L, Skaftnesmo K. O, Andersson E, Kleppe L, Edvardsen R. B, Norberg B, Fjelldal P. G, Hansen T. J, Schulz R. W, Wargelius A

**Affiliations:** ^1^ Research Group Reproduction and Developmental Biology, Institute of Marine Research, Bergen, Norway; ^2^ Embrapa Amazonia Ocidental, Manaus, Brazil; ^3^ Reproductive Biology Group, Department Biology, Science Faculty, Utrecht University, Utrecht, Netherlands

**Keywords:** germline, CRISPR/Cas9, spermatogenesis, argonaute protein (AGO), fish sterility

## Abstract

Genetic introgression of farmed salmon into wild populations can damage the genetic integrity of wild stocks and is therefore considered as an environmental threat. One possible solution is to induce sterility in farmed salmon. We have searched for proteins potentially essential for germline survival in Atlantic salmon. One of these is the argonaute protein Piwil1, known to be required for germ cell survival. To examine Piwil1 function in salmon, we induced indels in the N domain by CRISPR-Cas9. The encoded domain is present in all vertebrate Piwi proteins and has been linked to Tdrd1 protein interaction and PAZ lobe structure. The F0 founder generation of *piwil1* crispant males and females displayed a mosaic pattern of *piwil1* mutations, exhibiting highly mutated alleles (53%–97%) in their fin gDNA samples. In general, *piwil1* crispants carried germ cells, went through puberty and became fertile, although a transient and partial germ cell loss and delays during the spermatogenic process were observed in many male crispants, suggesting that Piwil1 functions during salmon spermatogenesis. By crossing highly mutated F0 founders, we produced F1 fish with a mixture of: loss-of-function alleles (^−^); functional in frame mutated alleles (^*+*^) and wt alleles (^+^). In F1, all *piwil1* ^*−/−*^ fish lacked germ cells, while *piwil1* ^*+/+*^ siblings showed normal ovaries and testes. Yet, most juvenile F1 *piwil1* ^*+/*−^males and females displayed an intermediate phenotype with a higher somatic/germ cell ratio without an increase in germ cell apoptosis, suggestive of a gene dose effect on the number of germ cells and/or insufficient replacement of lost germ cells in heterozygous fish. Interestingly, the two longest in-frame indels in the N domain also ensured germ cell loss. Hence, the loss of 4–6 aa in this region *Phe130*-*Ser136* may result in crucial changes of the protein structure, potentially affecting piRNA binding of the PAZ lobe, and/or affecting the binding of Piwil1 interacting proteins such as Tdrd protein, with critical consequences for the survival of primordial germ cells. In conclusion, we show that loss of *piwil1* leads to loss of germ cells in salmon and that part of the N domain of Piwil1 is crucial for its function.

## Highlights

• Loss of *piwil1* leads to loss of germ cells in salmon.
• Transient disturbances of spermatogenesis in F0 crispants suggest a function of Piwil1 during salmon spermatogenesis.
• The loss of 4 or 6 amino acids in the N domain (Phe130-Ser136) critically affects salmon Piwil1 function and leads to germ cell loss, potentially interfering with the binding of Piwi interacting proteins, such as Tdrd1, and/or the piRNA binding properties of the PAZ lobe.

## Introduction

This study aimed to investigate the function of the germ cell-specific argonaute protein Piwil1 (P-element induced wimpy testis) in Atlantic salmon (*Salmo salar* L.), with the long-term goal to develop a method to induce sterility in farmed fish. This would avoid genetic introgression of escaped farmed fish in wild populations (Glover et al., 2013), and the reduced welfare and harvest quality in farmed fish caused by un-wanted sexual maturation (Taranger et al., 2010). In vertebrates, Piwi proteins are mainly expressed by germ cells, where they bind their own class of small RNAs (piRNAs) forming an active riboprotein complex that recognizes and suppresses transposon activity, allowing the proper development and establishment of the germline (Cox et al., 2000; Iwasaki et al., 2015; Ozata et al., 2019; Yamaguchi et al., 2020; Ramat and Simonelig, 2021). Recent studies on the crystal structure of Piwi revealed that it consists of four domains (N, PAZ, MID, and PIWI) and three linker regions (L0, L1, and L2), and that it can be divided into two lobes (N-PAZ and MID-PIWI) (Yamaguchi et al., 2020). The PAZ domain recognizes and binds to the 3′ end of piRNAs (Lingel et al., 2003; Yan et al., 2003). The N-domain, the three linker regions, and the PAZ domain jointly constitute the N-PAZ lobe, which contributes to the stability of the guide-target duplex. The N-PAZ lobe also contains a binding site for Tudor domain (Tdrd) proteins, which are essential for the trimming of piRNA 3’ ends and germplasm assembly when bound to *piwil1* (Huang et al., 2011; Doxzen and Doudna, 2017; Ding et al., 2019).

Most mammals have four PIWI proteins (PIWIL1, PIWIL2, PIWIL3, and PIWIL4), with the exception of mice which lack Piwil3 (Carmell et al., 2007; Unhavaithaya et al., 2009). Fish in general only have two Piwi proteins, such as Ziwi and Zili in zebrafish (*Danio rerio*), Olpiwi1 and Olpiwi2 in medaka (*Oryzias latipes*) (Houwing et al.,. 2007; Houwing et al.,. 2008; Zhao et al., 2012), Piwil1 and Piwil2 in Atlantic salmon (Kleppe et al., 2015) and sturgeon (*Acipenser dabryanus*) (Yang et al., 2020). Loss of function studies in mammals and fish suggest that some functions of Piwi proteins on male germ cell development are conserved across different species. In mouse, loss of *Miwil1* leads to spermatogenic arrest at the beginning of the round spermatid stage and male sterility (Deng and Lin, 2002), and *Miwil2* or *Mili* knockout (KO) results in an arrest at the early prophase of the first meiotic division and also male sterility (Kuramochi-Miyagawa et al., 2004; Carmell et al., 2007), while female fertility remains intact. In zebrafish, loss of *ziwi* has no effect on the number of germ cells in embryos, but at around 3 weeks of age, z*iwi* ^−/−^ males lose all spermatogonia stem cells due to apoptosis at the beginning of pubertal spermatogenesis, indicating the inability of spermatogonial stem cell to differentiate in the absence of Ziwi (Houwing et al., 2007). Loss of *zili* increases transposon transcripts, and the type B spermatogonia increasingly lose the ability to differentiate, ultimately leading to the loss of these cells and sterility around 7 weeks of age in young adult males (Houwing et al., 2008). However, in the golden hamster (*Mesocricetus auratus*), which has 4 PIWI proteins, *Piwil1* KO males lack germ cells and females display non-functional oocytes (Hasuwa et al., 2021). In medaka, morpholino-mediated knockdown of *olpiwi* leads to complete loss of primordial germ cells (Li et al., 2012). It is uncertain how loss of *piwil1* in other fish species affects germ cell survival and development.

We have previously studied the two *piwi* paralogs in the Atlantic salmon genome and found that *piwil1* is highly and exclusively expressed in the germ cells of embryos and juvenile fish, while *piwil2* is expressed at a low level only in juvenile gonads (Kleppe et al., 2015). Hence, hypothesizing that *piwil1* is the paralog protecting the salmon germline, we targeted the N domain of *piwil1* in salmon using CRISPR/Cas9, to study the functionality of Piwil1 in salmon. Due to the long generation time in salmon (3 years), maturation was stimulated on highly mutated albeit mosaic F0 salmon using environmental triggers. F0 males were able to mature and produce sperm, although showing partial, transient germ cell loss and delays during spermatogenesis, and F0 females (not sampled during maturation) went through sexual maturation and became fertile. In the F1 generation, all *piwil1* ^*−/−*^ F1 fish lacked germ cells, and *piwil1* ^*−/+*^ F1 fish showed gonads with a reduced number of germ cells. Interestingly, we found that the longest in-frame mutations in the N domain also led to loss of function, suggesting that losing 4–6 amino acids in this region of the N domain is incompatible with Piwil1 functionality.

## Materials and methods

### Guide RNA design and synthesis

High scoring guide RNA (gRNA) target sequences were predicted using CRISPRscan (Moreno-Mateos et al., 2015). Templates for producing gRNAs were then made according to protocols published by Gagnon and co-workers (Gagnon et al., 2014) with minor modifications as outlined in our previous publication (Skaftnesmo et al., 2021). The guide used to target *piwil1*; AGG​CGC​CGA​GAC​TCC​ATG​ACG​GG, locates to chromosome 24, NC_027323.1. The targeted region corresponds to nucleotide 616-638 in the encoded *piwil1* mRNA (XM_014171837), an area close to the encoded aa132 in the N domain. Cas9 mRNA was prepared as previously described (Edvardsen et al., 2014).

### Microinjection and embryo rearing

Microinjection of about 1,500 freshly fertilized Aquagen strain embryos was carried out in November 2015, and took place as previously described (Edvardsen et al., 2014). Two gRNA´s were used; targeting *piwil1* and *slc45a2*, which were co-injected with mRNA encoding Cas9. We targeted *slc45a2* in addition to *piwil1*, as previous work has shown that the resulting phenotype, i.e., loss of pigmentation, is a reliable indicator of the efficiency of a co-injected, second gRNA (Wargelius et al., 2016). About 500 uninjected Aquagen strain control embryos served as control fish later in the study. The embryo containers were in the same tray at 6°C, until they started feeding, at Matre Aquaculture Research Station (Matredal, Norway). Full or partial albinos as well as control (non-injected) alevins were collected, start fed from March 2016, and reared in common garden under standard conditions until sorting in September 2016 (see below).

### Piwil1 crispant F0 rearing

At 10 months of age (September 2016), *piwil1* crispants displaying partial or full loss of pigmentation (*n* = 147) were kept together with an equal number of control (pigmented) fish. Five fish displaying an albino phenotype were Sanger sequenced to confirm CRISPR induced mutation in the *piwil1* gene. Primers and methods for this analysis are detailed below in the section describing mutational analysis of the target site. At 16 months of age, *piwil1*-crispants were fin clipped and PIT-tagged for identification. All crispants were *sdy* assayed to determine the genetic sex (Ayllon et al., 2019) and found 69 males and 78 females in this group of crispants. This enabled targeted sampling of either males or females.

### Piwil1 crispant F0 males, post smolt maturation regime and samplings

On 30 January 2017, a postsmolt maturation regime started in freshwater, by exposing 14 months old fish to 16°C and continuous light for 6 weeks to stimulate early male maturation (Fjelldal et al., 2011). Fish were then transferred to brackish (25 ppm) water and reared at ambient temperature onwards. On January 24th, February 21st, May 9th and 26 September 2017, samplings took place from crispant and wt groups. Gonad and blood samples were collected, and gonado-somatic indices (GSI) calculated from crispants and wt fish. A small fragment of each gonad was fixed in 4% glutaraldehyde for plastic embedding (Technovit 7100; Kulzer) and histological analyses, as previously described (Wargelius et al., 2016; Kleppe et al., 2017).

F0 females were not sampled to study ovarian growth and maturation because it is not possible to trigger maturation in females as described above for males. Moreover, females are more sensitive than males to disturbances during gonad maturation. Instead we waited one more year, and in autumn 2018 females were maturing.

### 11-KT quantification by ELISA

The levels of 11-ketotestosterone (11-KT) were analyzed by ELISA (Cuisset et al., 1994) on extracted plasma samples from males, as previously described (Andersson et al., 2013). Acetylcholine esterase-labeled tracers and microplates pre-coated with monoclonal mouse anti-rabbit IgG were supplied by Cayman Chemicals. Anti-11-KT was a kind gift from David E. Kime (Sheffield University, United Kingdom).

### Crossing of F0 piwil1 crispants

F0 *piwil1* crispants of both sexes were reared in brackish water (25 ppm) during the winter 2017/2018, and were transferred to freshwater in June 2018. In September 2018, all visually immature *piwil1* crispant and control fish were separated from the visually mature fish and in September 2018, all immature *piwil1* crispants were terminated and sampled to confirm the immature stage of their gonads. In November 2018, we sampled the tank which contained the visually mature fish. In these fish we measured size, opened and registered maturation in F0 crispants females, except for a few fish kept for breeding purposes (see below). A total of 14 *piwil1* crispant mature males and 7 *piwil1* crispant mature females, were deep sequenced at the *piwil1* gRNA target site, with the aim to estimate mutation types and level of knockout in F0. Four highly *piwil1* mutated mature broodstock F0 fish, two males and two females, were chosen for crossings to produce an F1 generation in November-December 2018.

### Piwil1 F1 mutants’ maintenance and samplings

*Piwil1* F1 hatched in March 2019 and were maintained at 6°C until start feeding in May 2019, from when they were kept at 13°C and continuous light for 3 months. In August 2019, fish were transferred to ambient conditions until sampling, i.e., were not exposed to a maturation regime. A total of 129 F1 fish as well as 53 wt (pigmented) fish of the same age, all raised in a common garden sibling settings, were sampled six times (August 28th and December 17th in 2019; May 14th, September 9th and 16th in 2020; and January 29th of 2021), until reaching 2 years of age, since we wanted to examine a possible progressive loss of germ cells. Gonads were collected and weighted for estimation of the GSI. A small fragment was rapidly immersed in RNA later for total RNA isolation and qPCR analysis, and another piece immediately fixed in Bouin’s solution or in 2.5% buffered glutaraldehyde and included in paraffin or methacrylate for histology, according to routine procedures. A fin clip was also collected for genotyping for *piwil1* mutations.

### Mutational analysis of target site

DNA purified from *piwil1* crispant fish was amplified in a two-step nested barcoding PCR. A forward primer: 5′-TCT TTC CCT ACA CGA CGC TCT TCC GAT CTG ATG CAG GAA TCA ACA CCA GGC-3′ and reverse primer: 5′-TGG AGT TCA GAC GTG TGC TCT TCC GAT CTT TTG TGA GGC AGG AAG AGG AGG-3′ spanning the CRISPR targeted region were used in the initial PCR cycles. The amplicons were then subjected to a nested PCR with barcoding primers as described in Gagnon et al. (2014). The final denatured sequencing library was prepared at a concentration of 8 p.m. and spiked with 5% denatured phiX and sequenced on the MiSeq using MiSeq Reagent Kit v3 (600 cycle format). Analyses of the FASTQ sequences were performed using CRISPResso2 (Clement et al., 2019) as previously described in Skaftnesmo et al. (2021). For the individuals that were Sanger sequenced (both F1 and *piwil1* crispant fish), PCR amplicons were generated using forward primer; CAG​AGG​AAT​AGT​GCC​CTC​CA, and reverse primer; CCT​TTG​ATA​TTT​CCT​CAG​GT in a PCR reaction containing Q5 polymerase (NEB) according to the manufacturer’s recommendations. PCR products were then treated with ExoSAP-IT (Thermo Fisher), and 1 µl of the ExoSAP treated PCR products were used as input in a Sanger sequencing reaction (Big-Dye version 3.1) with 3.2 pmol of the sequencing primer; CCT​TTG​ATA​TTT​CCT​CAG​GT according to the manufacturer’s recommendations. Sanger traces were decomposed using Synthego Inference of CRISPR Edits (ICE) in order to evaluate CRISPR efficiency and types of edits (Conant et al., 2022).

### RNA extraction and cDNA preparation

Small fragments of testis and ovaries sampled from the F1 group were stored in RNAlater. A fragment of approximately 50 mg was homogenized in 400 µl of the homogenization buffer and processed according to the Maxwell HT-simplyRNA kit instructions (Promega) on a BioMek 4000 instrument (Beckton Dickinson). The quantity and purity of RNA samples were further assessed by spectrophotometry on a nanodrop ND-1000 instrument (ThermoFisher Scientifics). cDNA was prepared by reverse transcription of 200 ng RNA using the SuperScript IV VILO Master Mix with ezDNase enzyme according to the manufacturer’s recommendations (Thermo Fisher Scientific).

### Quantitative PCR

All qPCR assays were previously published (*vasa, gsdf, cyp19a1a, amh*; Skaftnesmo et al., 2021). A qPCR reaction was prepared to contain 800 nM of each forward and reverse primer, 250 nM of the probe in a 6 µl reaction containing 1x concentration of the TaqMan Fast Advanced Master Mix (Thermo Fisher Scientific) and 2 µl of a 1/20 diluted cDNA. The reaction was subjected to thermocycling in a QuantStudio 5 Real-Time PCR system (Thermo Fisher Scientific) with an initial hold at 50°C for 2 min followed by an initial denaturation step at 95°C for 2 min. Thermocycling was conducted for 40 cycles using a denaturation step at 95°C for 1 s followed by a combined annealing and extension step at 60°C for 30 s. Data was processed at Thermo Fisher cloud using the relative quantification app. QPCR was performed in duplicates in 384-well optical plates in a QuantStudio 5 Real-Time PCR system (ThermoFisher Scientific). No-template controls for each gene were run in all qPCR plates. The relative gene expression level was calculated using the 2−ΔΔCt method. All values were normalized to *ef1a* and calibrated to the average ΔCt of the controls of each sex.

### Homology modeling

The structure of salmon Piwil1 was modeled by homology modeling to the closest resembling structure (7kx9.1) using Swissmodel (Waterhouse et al., 2018). The modeled protein structures were then imported into ChimeraX (Pettersen et al., 2021), aligned to the reference model and colored to highlight changes in overall structure using the positional root-mean-square deviation (RMSD) between two sets of atoms in the mutant versus wildtype.

### Statistical analysis

All data were checked for normal distribution and homogeneity of variance by the Kolmogorov-Smirnov and Levene’s test, respectively. Data on gene expression (qPCR) was log transformed before testing their equality by ANOVA, followed by Tukey post hoc test, where three groups were compared. In the graphs with only two groups, the significant differences in their raw data was identified using Student’s t test. For comparing the GSI of F0 crispants, ANOVA was used, followed by Tukey test. Data are represented as mean ± SEM. GraphPad Prism 8 (GraphPad Software, Inc.) was used for statistical analysis.

## Results

### Piwil1 F0 crispant salmon

**FIGURE 1:**
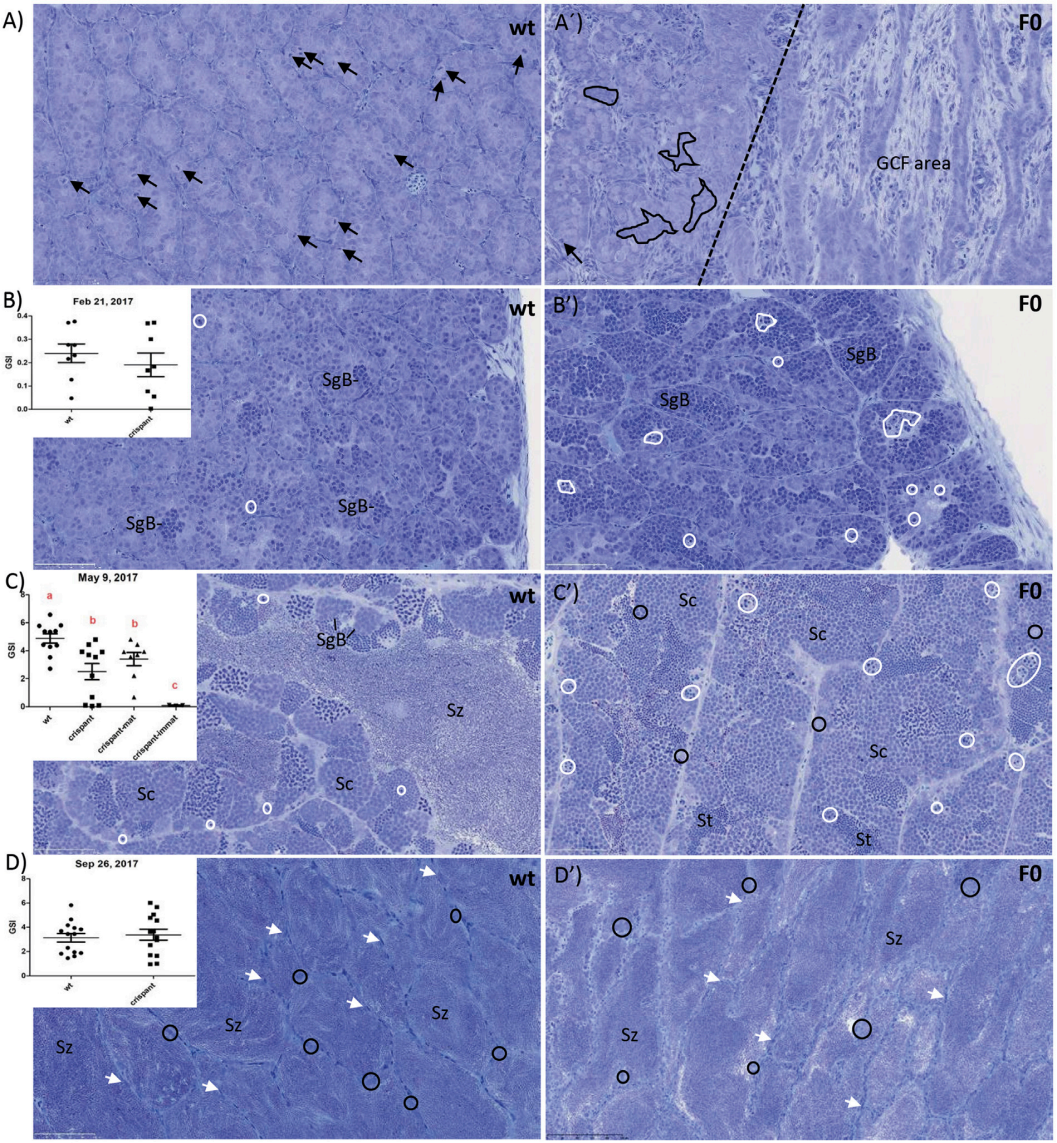
Gonadosomatic index (GSI; %) and testis histology of wild type (wt) and piwil1 F0 crispant Atlantic salmon: **(A)** 24 January 2017: males of both genotypes were immature, as indicated by spermatogenic tubules containing only Sertoli cells and type A spermatogonia. The testes of 3 out of 5 crispants showed germ cell-free areas containing only Sertoli cells (see area to the right of the stippled line). The apparent frequency of cells in mitosis (black arrows) was lower in crispant testes that also showed several areas in which Sertoli cell groups were not in contact with germ cells (black delineated areas in A′); **(B)** 21 February 2017: puberty had started in both genotypes, as indicated by the presence of type B spermatogonia (SgB); apoptotic cells (white delineated areas) were found frequently in crispant testes but rarely in wt testes; **(C)** 9 May 2017: rapid testicular growth phase reflecting full spermatogenic activity, maturing crispants showed a lower GSI, apparently less spermatozoa (Sz) and apoptotic cells (white delineated areas) than wt males. **(D)** 26 September 2017: the large majority of both wt and crispant males were spermiating 599 and the lumina of the spermatogenic tubules were filled with spermatozoa (Sz), while the germinal 600 epithelium consisted of a single layer of Sertoli cells (white arrows) and scattered single, apparently 601 quiescent type A spermatogonia (black circles). Black arrows: mitosis; white-delineated areas: 602 apoptotic germ cells; white arrows: Sertoli cells. Immat—immature, mat—maturing. Sc—603 spermatocyte, SgB—type B spermatogonia, St—spermatid, Sz—spermatozoa.

**FIGURE 2:**
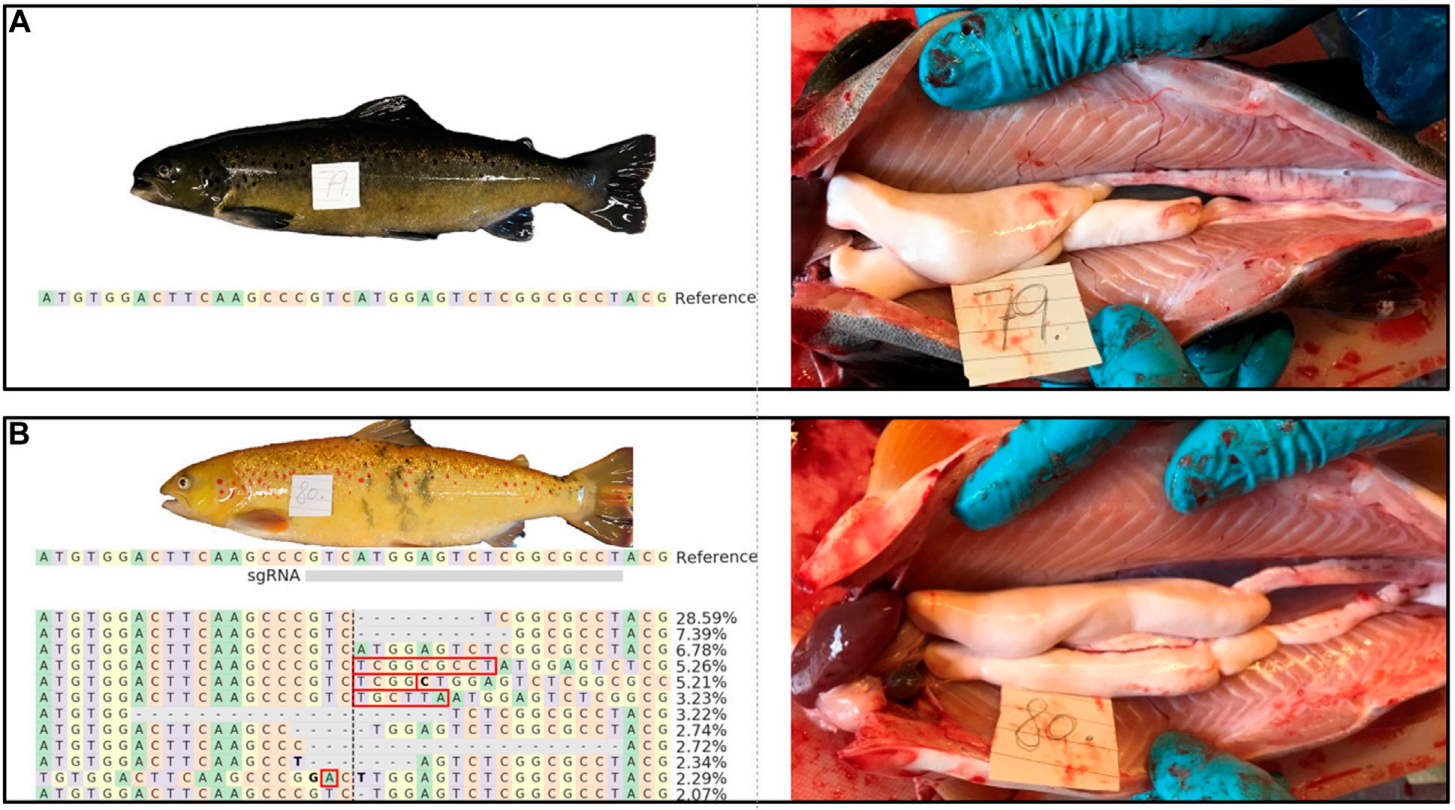
External appearance and macroscopic testis anatomy of 1 year old wt [**(A)**; animal #79) and *piwil1* F0 crispant Atlantic salmon [**(B)**; animal #80) males. The table in B shows an alignment of the most common (up to 2%) *piwil1* mutations found in #80. Red rectangles show inserted bases and bold letters indicate nucleotide substitutions.

All F0 *piwil1* crispants fish were viable, and mortality and visual deformity rates did not differ from the control (wt) group. Initial Sanger decomposition analysis of the *piwil1* gRNA target site in a subset of five F0 *piwil1* crispants revealed high mutation inducing activity (data not shown). To run a maturation experiment on *piwil1* crispant males, *piwil1* crispants and wt males were exposed to a 6-weeks stimulatory photoperiod from January 30th to 15 Marc^h^ 2017. Between January 24th and September 26th, a total of 39 crispants and 43 wt males were sampled, dispersed on four occasions (January, February, May and September). Mean GSI of both groups was similar in February and September (due to a technical failure, gonad weight could not be determined in January). In May, when GSI values had increased at least ∼10-fold, reflecting the rapid, super-allometric testis growth based on full spermatogenic activity in both, wt and crispant maturing males, the mean GSI of maturing crispants was lower than that of maturing wt males (Figure 1C). The histological analysis of *piwil1* crispants and wt testis sampled in these four moments revealed that 56% of F0 crispants showed a testicular development similar to wt control males. However, the remaining crispants, found scattered at the four sampling dates, showed abnormalities, such as a completely or partially germ cell free testis (Figure 1A), abundant display of germ cell apoptosis (mainly during the rapid testicular growth phase from February to May; Figures 1B,C), or delays in the spermatogenic process, during the rapid super allometric testis growth in May (Figure 1C). The phenotypic heterogeneity among the F0 crispants probably reflects their genetic mosaicism regarding different *piwil1* mutations. Nevertheless, there was no difference between groups at spawning as 15 out of 16 and 17 out of 19 *piwil1* crispants and wt males, respectively, sampled in September, were spermiating (Figures 1J,K) and during sampling they showed “running milt” (Figure 2).

**FIGURE 3:**
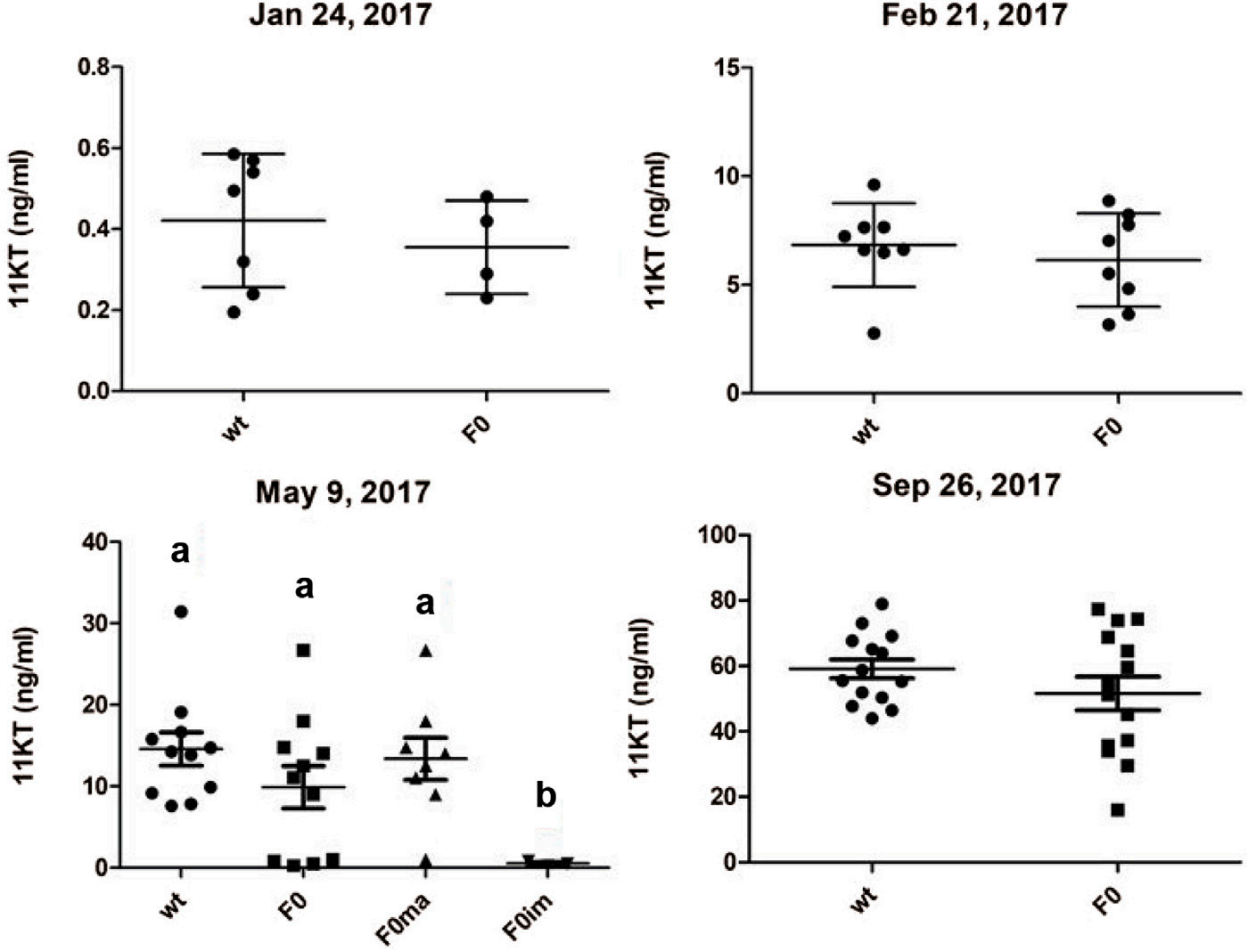
Plasma levels of 11-ketotestosterone (11KT; ng/ml) of 1 year old male Atlantic salmon (control - wt and *piwil1* F0 crispant). Ma-maturing, im–immature.

Measuring 11-ketotestosterone (11-KT) plasma levels throughout the maturation experiment showed that mean 11-KT levels did increase with the progressive maturation of the testis in *piwil1* F0 crispant and control males (Figure 3). Before and after the start of the maturation regime, wt and *piwil1* F0 crispant males did not show statistically significant differences in their 11-KT plasma levels. This included the maturing crispants sampled in May, although they showed a lower GSI level than maturing wt males (Figure 1C).

**FIGURE 4:**
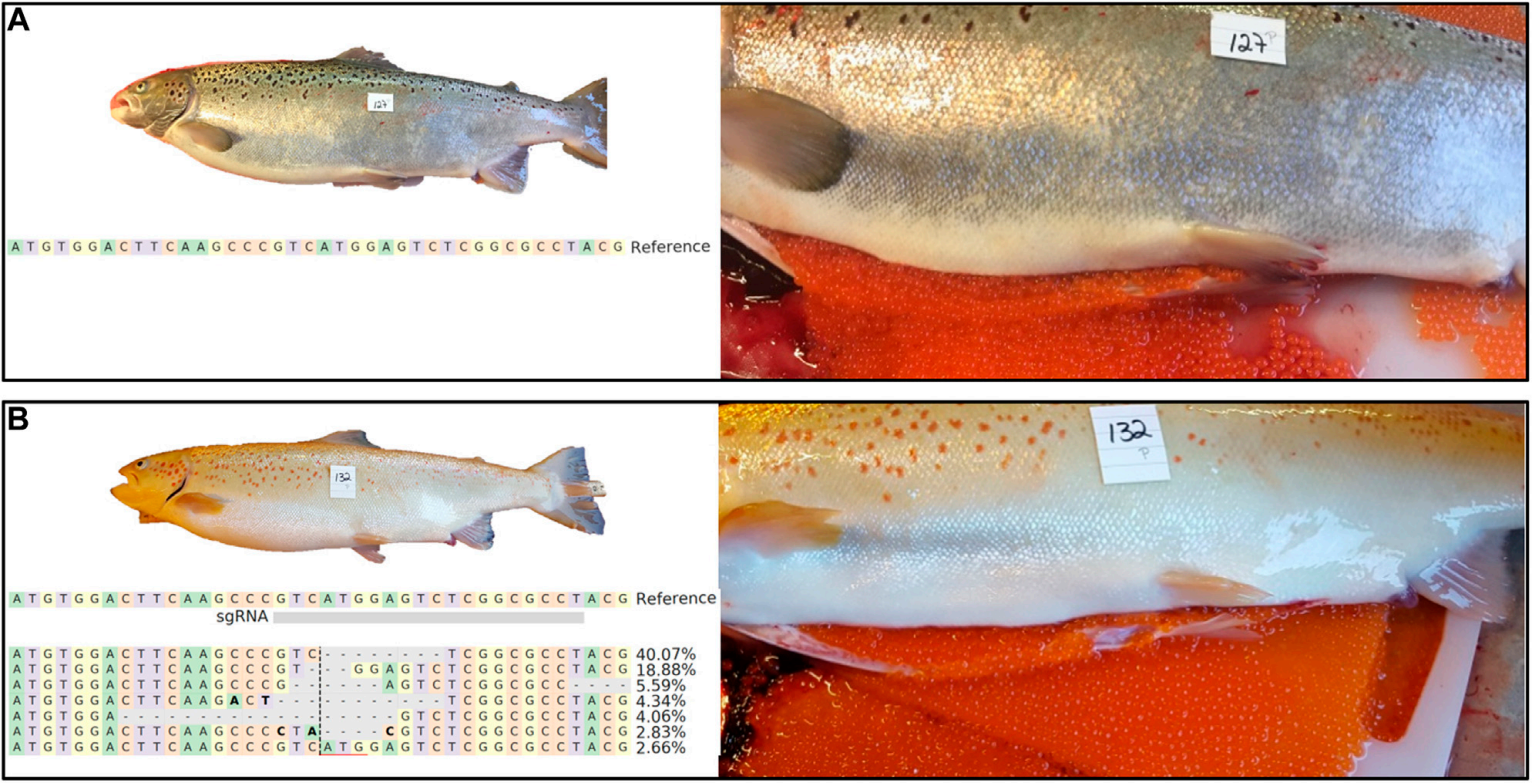
External appearance and macroscopic view of ovulated eggs of 2 years old wt [**(A)**; animal #127) and *piwil1* F0 crispant Atlantic salmon [**(B)**; animal #132) females. The table in B shows an alignment of the most common (up to 2%) *piwil1* mutations found in #132. Bold letters indicate nucleotide substitutions.

As expected, female *piwil1* crispants and controls required 1 year more than the stimulated males before ovarian maturation started in 2018. As female *piwil1* crispants were not sampled during maturation, we have no information on the possible effects of *piwil1* mutagenesis on ovarian maturation. However, in November 2018, 24% of the crispant females (*n* = 27) and 45% of the wt females (*n* = 26), had ovulated and could be stripped for egg collection. The gross morphology of the ovulated eggs was similar in wt and *piwil1* crispant females (Figure 4).

### *piwil1* F0 breeding

**FIGURE 5:**
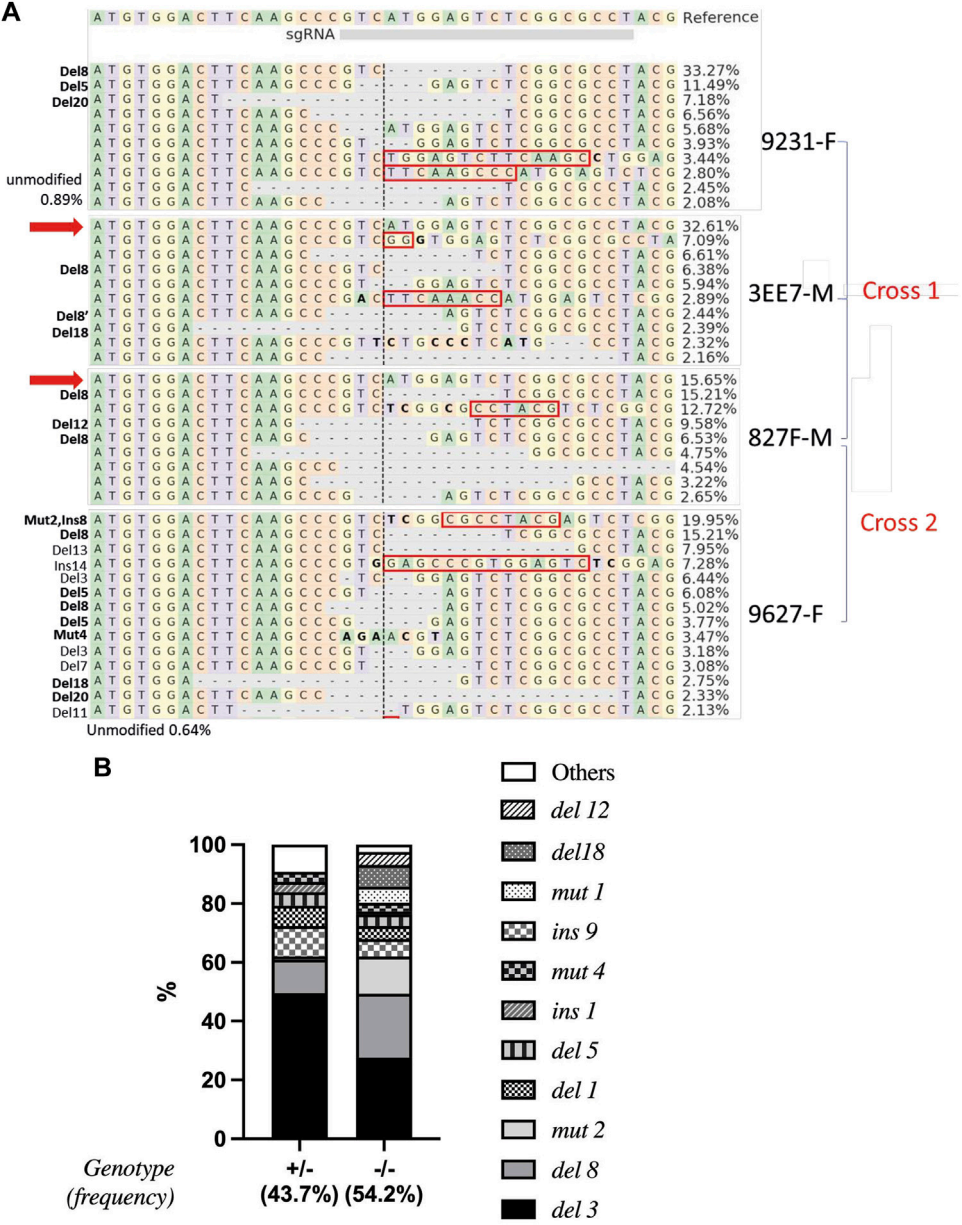
Alignment and frequencies of indels in *piwil1* F0 and F1 Atlantic salmon. **(A)** Alignment of the sequences identified in the *piwil1* crispant F0 salmon used as breeders to produce the F1 generation. In bold, indels that lead to loss of function in homozygosis. Red arrows point to wt *piwil1* copy (present only in the males). Indels with frequency lower than 2% were not included in the figure. Red rectangles show insertions and bold letters indicate base substitutions. **(B)** Frequency of indels found in *piwil1* ^+/−^ and *piwil1* ^−/−^ F1 salmon. F: female; M: male.

A total of 19 mature *piwil1* crispants F0 (7 females and 12 males) were analyzed by deep sequencing, to identify individuals suited for producing an F1 generation. This analysis revealed high mutation rates in the *piwil1* gene in all of *piwil1* crispant broodstock (53%–97%, Supplementary Table S1). From those, two males and two females were selected based on overall mutation rates. In December 2018, we made two crosses with these 4 F0 *piwil1* crispants. The mutation rate was high in all founders (Figure 5A).

We did not measure larval survival rate in crosses, however there were normal numbers of offspring in all crosses and all larvae were reared in a common garden. We sampled the F1 generation of *piwil1* mutants six times between 10 and 27 months of age.

### Mutation types and resulting gonad phenotypes in piwil1 F1 mutants

**FIGURE 6:**
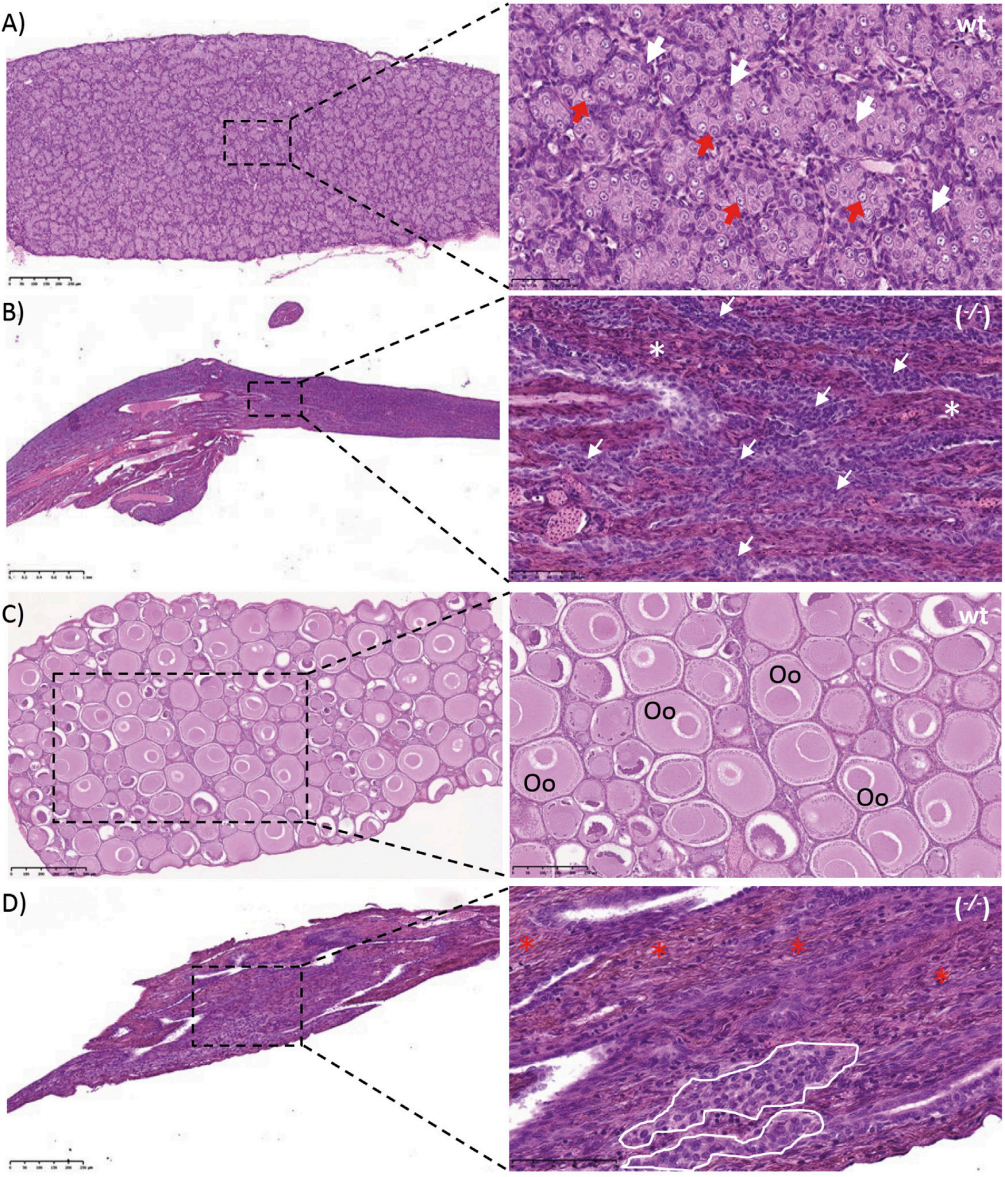
Gonads of wild type (wt) and *piwil1* F1 KO (^−/−^) Atlantic salmon. **(A)** Testis of a wt male. **(B)** Testis of a piwil1 KO (*del20/del5*) male (#298). **(C)** Ovary of a wt female. **(D)** Ovary of a piwil1 KO (*del8/del12*) female (#225). White arrows point to Sertoli cells; red arrows show type A spermatogonia; white asterisks mark dense interstitium and red asterisks mark interstitium rich in fibrocytes and extracellular matrix; white line marks groups of follicle cells. Oc: previtellogenic oocyte.

All sampled F1 fish were genotyped to determine the type of mutation in *piwil1*. 54.2% carried double allelic mutation (*piwil1* ^*mut/mut*^), 43.7% had one allele mutated (*piwil1* ^*wt/mut*^) and 2.1% were wild-type for *piwil1*. A total of 17 indel types were identified by Sanger sequencing, reflecting the mosaicism of the parents (Figures 5A,B). All double allelic loss-of-function (*lof*), *−/−* genotypes for *piwil1* resulted in GCF fish (Table 1; Figure 6), carrying any combination of the following nonsense mutations; *del1*, *del5*, *del8*, *del20*, and *ins1*, and the stop codon forming mutation; *mut4*. In addition, we observed that the combination of the nonsense allele *del8* and *in-frame* alleles *del12* and *del18* also resulted in GCF fish, indicating that these two *in-frame* deletion genotypes also resulted in non-functional alleles. On the other hand, the following *in-frame* mutations did seem to create functional alleles (*fmut*); *del3*, *ins9*, *mut1*, and *mut2,* as fish with these combinations had germ cells.

**TABLE 1:** Date, fish biometry, gonad phenotype and mutation type (sequences below) of *piwil1* mutant F1 salmon sampled from December 2019 to January 2021.

Date of sampling	Sample	Length	Weight (g)	Gonad weight (mg)	GSI	Gonad phenotype	Mutation
Dec. 2019	155	18.4	76	no gonad	0.000	F GCF	*del8/ins1*
Dec. 2019	157	17.8	64	no gonad	0.000	F GCF	*del8/mut4*
Dec. 2019	175	19.8	97	no gonad	0.000	F GCF	*del8/del18*
Feb. 2020	183	10.7	20.6	*		M GCF	*del8/del12*
Feb. 2020	187	12.0	22	*		F GCF	*del8/del18*
Feb. 2020	191	6.2	18	*		F GCF	*del8/del4*
May 2020	201	26	178	4	0.002	F GCF	*del20/mut4*
May 2020	203	24	150	23	0.015	M GCF	*del8/del8*
Sep. 2020	225	33.5	438	28	0.006	F GCF	*del8/del12*
Sep. 2020	268	35	479	no gonad	0.000	F GCF	*del8/del8*
Sep. 2020	273	32	356	62	0.017	M GCF	*del8/del8*
Sep. 2020	290	33	414	54	0.013	M GCF	*del8/del5*
Sep. 2020	295	28.5	258	52	0.020	M GCF	*del1/del1*
Sep. 2020	298	34	442	89	0.020	M GCF	*del20/del5*
Sep. 2020	308	33.5	475	77	0.016	M GCF	*del8/del1*
Sep. 2020	321	30	349	7	0.002	F GCF	*mut4/mut4*
Sep. 2020	324	33	462	10	0.002	F GCF	*del8/del8*
Jan. 2021	341	40	825	46	0.006	F GCF	*del20/del8*
Jan. 2021	342	47.5	1,436	325	0.023	M GCF	*del18/del8*
Jan. 2021	343	46	1,349	76	0.006	F GCF	*del18/del8*
Jan. 2021	344	45.5	1,214	87	0.007	F GCF	*del12/del8*
Jan. 2021	345	44	1,101	76	0.007	F GCF	*del12/del8*
Jan. 2021	350	46	1,238	62	0.005	F GCF	*del12/del8*
Jan. 2021	358	42	913	602	0.066	M GCF	*del18/del8*
Jan. 2021	359	40	866	65	0.008	F GCF	*del18/del8*

In red, mutation that does not follow the pattern of the group. F GCF, female germ cell free, M GCF, male germ cell free, M Abnormal, male presenting testis with germ cell, but with some abnormality. *, we couldn’t sample the gonad due to its very small size.

**FIGURE 7:**
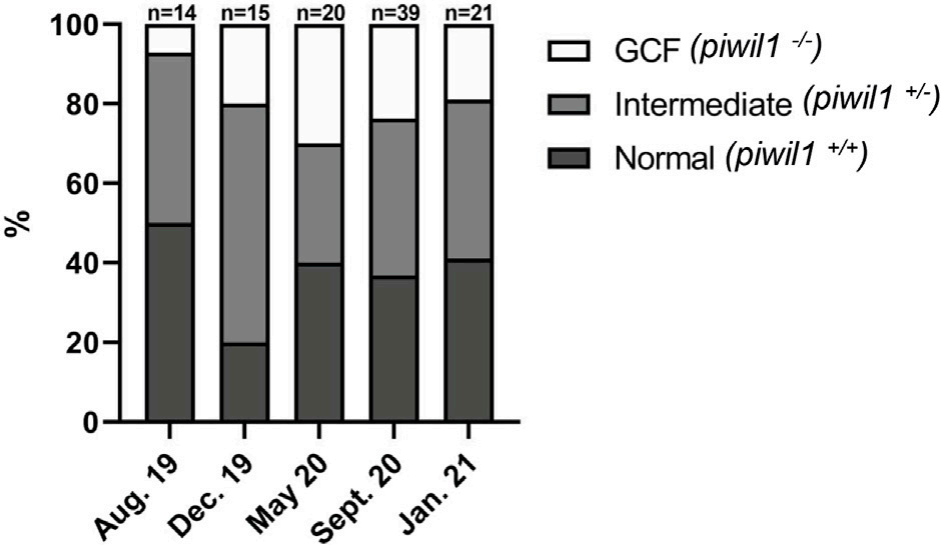
Phenotype and genotype of all *piwil1* F1 Atlantic salmon sampled on six occasions. Fish displayed germ cell free gonads (GCF), intermediate phenotype or normal gonads. Data from September 2020 combines two samplings in the same month (9th and 16th).

The GCF *piwil1* ^*−/−*^ testis presented a dense interstitium and Sertoli cells as the only cell type in the intratubular compartment, while the GCF *piwil1* ^*−/−*^ ovary displayed tissue rich in fibrocytes and extracellular connective tissue elements (Figure 6). Rare groups of follicle-like cells (distinct from connective tissue cells) were found scattered in the GCF ovaries. QPCR analysis for *vasa* of wt and *piwil1* ^*−/−*^ ovaries and testes confirmed the histological observations that germ cells were absent in *piwil1* ^*−/−*^ fish (Supplementary Figure S1). The 25 GCF *piwil1* ^*−/−*^ gonads were found in both sexes at all six sampling dates, and the GCF phenotype was consistent throughout all samplings, indicating that there was no progressive germ cell loss in *piwil1* ^*−/−*^ gonads (Figure 7).

**FIGURE 8:**
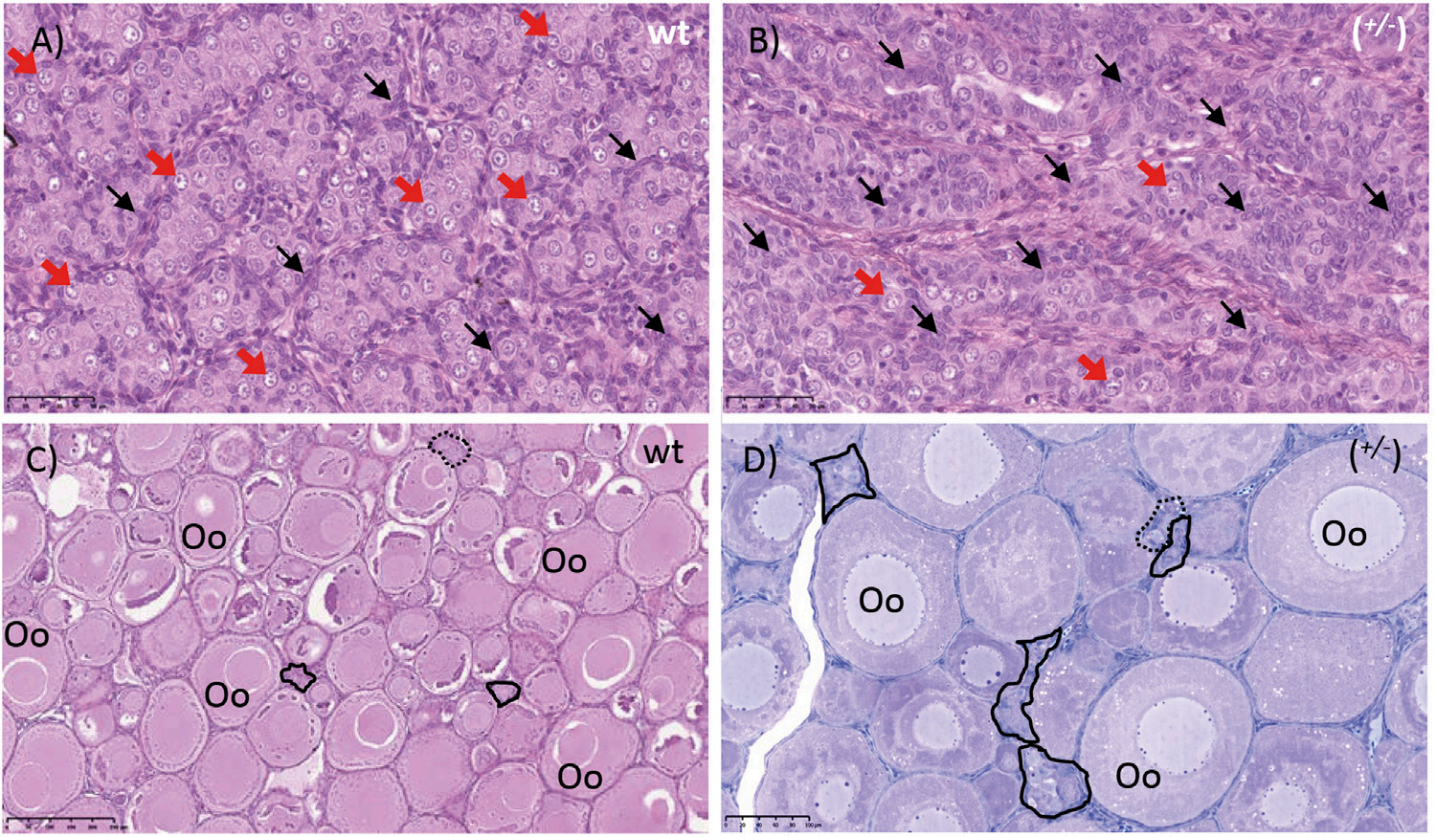
Gonads of *piwil1* ^+/+^ (wt) and *piwil1* ^+/−^ F1 Atlantic salmon. **(A)** Testis of a wild type (wt) male. **(B)**Testis of a *piwil1* ^+/−^ (*wt/del8*) male presenting a lower GC/somatic cells ratio. **(C)** Ovary of a wt female. **(D)** Ovary of a *piwil1* ^+/−^ (*del3/del8*) female with excess of early germ cells. Black arrows point to Sertoli cell nuclei; red arrows show nuclei of type A spermatogonia. Continuous line marks groups of oogonia and stripled line marks cystic oocytes. Oo: previtellogenic oocyte.

**FIGURE 9:**
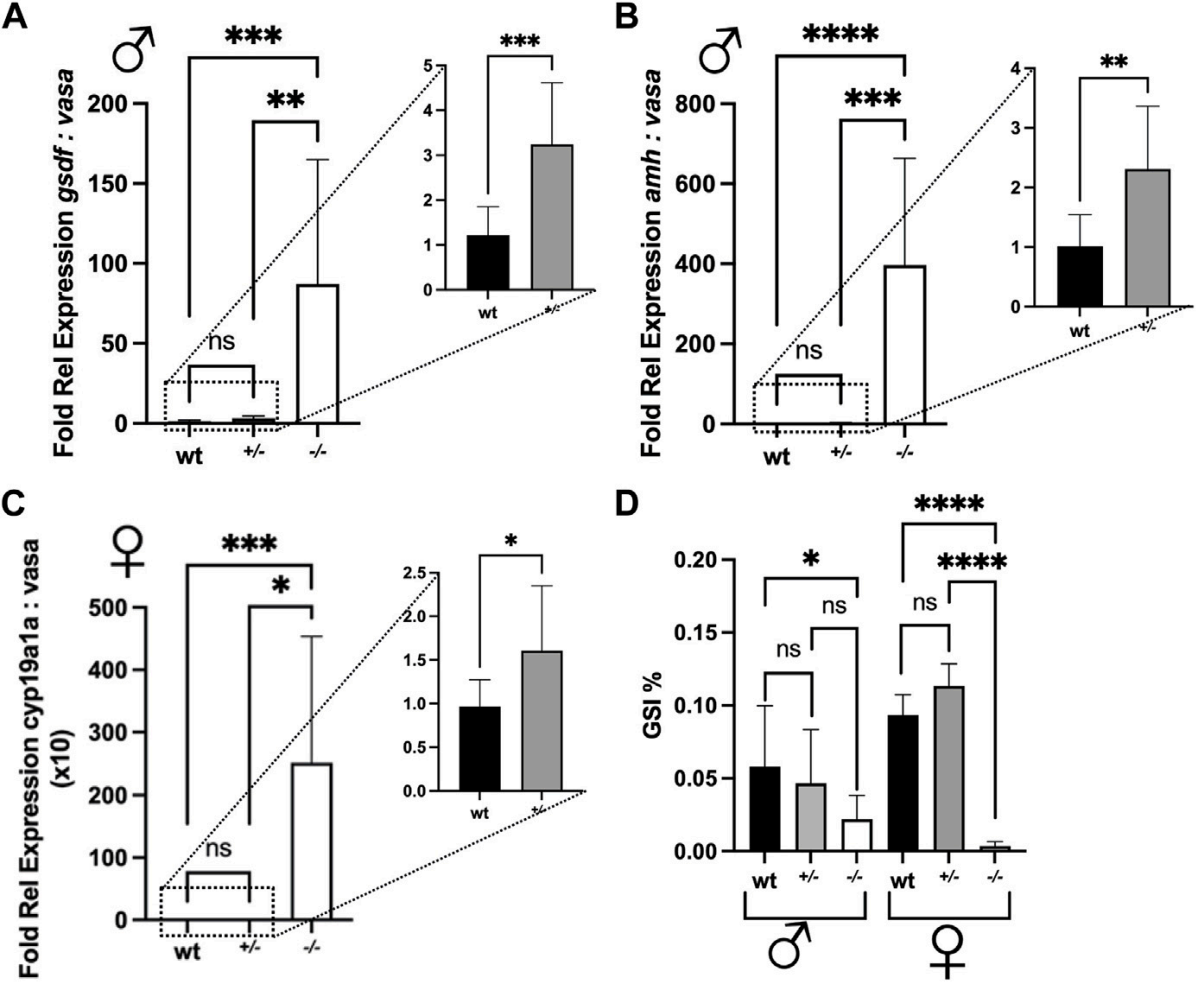
Fold relative expression of genes expressed in the gonads and gonadosomatic indices of wild type (wt), *piwil1* ^+/−^ and *piwil1* ^+/−^ F1 Atlantic salmon. **(A)** *gsdf* and *vasa* in testes. **(B)** *amh* and *vasa* in testes. **(C)** *cyp19a1a* and *vasa* in ovaries. **(D)** Gonadosomatic index (GSI) in wt, *piwil1* ^+/−^ and *piwil1* ^−/−^ F1 Atlantic salmon males and females.

Interestingly, considering heterozygous animals with one of the *piwil1 lof* alleles, all *piwil1* ^*+/−*^ fish presented an intermediate phenotype. Among these intermediate phenotypes, we found two additional nonsense mutations; *del11* and *del17*, which were not present among the GCF fish. The main intermediate phenotype observed in *piwil1* ^*+/−*^ males was a clear shift in the germ/Sertoli cell ratio in favor of Sertoli cells (Figure 8B). Another testis abnormality less frequent in *piwil1* ^*+/−*^ males was the presence of amorphous areas and/or the presence of immune cells in the parenchyma (data not shown). Different from F0 crispants, the incidence of germ cell apoptosis in testis tissue of heterozygous F1 males was as low as in wt fish, and the mean GSI value was also similar to those of the wt males (Figure 9D). In *piwil1* ^*+/−*^ females, we observed an apparent increase in the frequency of early oocyte cysts compared to wt ovaries, indicative of a delay on the transition from cystic oogonia/oocytes to previtellogenic oocytes individualized in follicles (Figure 8D). Still some *piwil1* ^*+/−*^ F1 females also showed an increased cellularity in the interstitium (data not shown). Means GSI of F1 and wt females were similar (Figure 9D).

The shift in the ratio between germ and Sertoli cells was then confirmed by comparing the ratios between *vasa* and *gsdf* or *amh* transcript levels, a germ and two Sertoli cell markers, respectively, in wt, *piwil1* ^+/*−*^ and *piwil1* ^−/−^ testes (Figures 9A,B). For the females, the ratio between *vasa* and *cyp19a1a* transcripts was also assessed by qPCR in wt, *piwil1* ^+/*−*^ and *piwil1* ^−/−^ ovaries (Figure 9C). *Piwil1* ^−/−^ fish presented the highest ratio in somatic: germ cell gene expression, as *vasa* expression was close to the detection limit. In spite of showing similar mean GSI values to wt siblings (Figure 9D), *piwil1* ^+/-^ males and females displayed a significantly higher expression ratio between somatic: germ cell genes compared to wt siblings, confirming the histology results of: 1) less germ cells in mutant males carrying only one *lof* allele (*piwil1* ^+/*−*^); and 2) more early germ cells (higher levels of *vasa* transcripts) in the *piwil1* ^+/*−*^ females.

**FIGURE 10:**
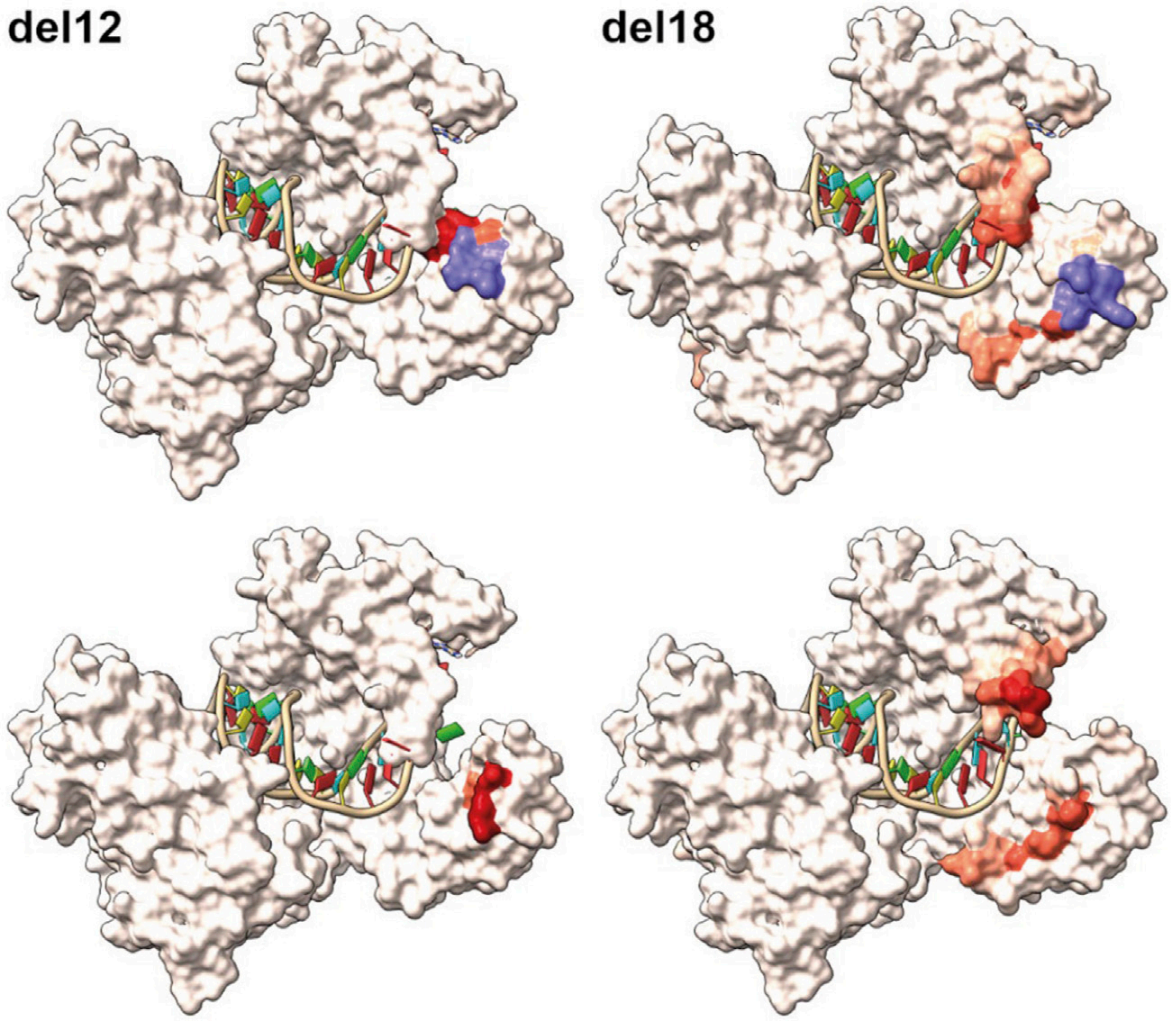
Homology modelling of the inframe *lof* alleles *del12* and *del18*. Top row displays the wildtype modelled salmon Piwi1 with deleted amino acids shown in purple. The red coloration is reflecting the positional root-mean-square deviation (RMSD) between two sets of atoms in the mutant versus wildtype. The bottom row displays the modelled structure of the variants with the red coloration indicating the RMSD value.

We also found some *in-frame* allele combinations in the F1 generation, which resulted in the loss of germ cells (*del12* and *del18*), indicating that the N-terminal region of Piwil1 encompasses an area critically sensitive to losing the following 4–6 amino acids: VMES, *Val133-Met134-Glu135-Ser136,* and DFKPVM, *Asp129-Phe130-Lys131-Pro132-Val133-Met134*. The N-domain targeted in our experiments is localized in a region conserved among vertebrates, situated in a flexible region between an alpha helix and a beta sheet and in close proximity to both. To estimate the potential effect of the loss of these amino acids, we predicted the tertiary structure of the *del12* and *del18* mutants by homology modeling with Swissmodel (Figure 10). Accordingly, both deletions seemed to cause structural changes by dislocating amino acids to protrude to the surface of the N-domain in proximity to the piRNA binding groove and close to the N-terminal protein binding domains of *piwil1*.

## Discussion

Approaches for inducing sterility by biotechnological methods in farmed fish include vaccination (Sambroni et al., 2009), gene knockdown (Wong and Zohar, 2015), surrogate production (Nagasawa et al., 2019) or gene knockout (Wargelius et al., 2016; Güralp et al., 2020). Here, in an attempt to produce sterile fish, we knocked out *piwil1* in salmon, since it is expressed exclusively in salmon germ cells (Kleppe et al., 2015) and is essential for germ cell survival in young adult male zebrafish (Houwing et al., 2007) and for primordial germ cell migration in medaka (Li et al., 2012). In the F0 generation, produced in 2015, high mutation rates in the *piwil1* gene were detected in fin-clips and by visual inspection of the co-targeted *slc45a2* gene, which produces an albino phenotype (Edvardsen et al., 2014)*.* Since *piwil1* is exclusively expressed in germ cells in salmon, we expected no direct effects of *piwil1* knockout (KO) in somatic cells, which was confirmed when analyzing both F0 and F1 generations of *piwil1* KO fish. Similar to findings in *dnd* KO salmon (Wargelius et al., 2016), we found that loss of *piwil1* leads to complete loss of germ cells in Atlantic salmon. Hence, Piwil1 protein can be an alternative target for induction of sterility in Atlantic salmon, especially if rescue methods of genetically sterile individuals can be further developed using several alternative proteins (Güralp et al., 2020).

The high mutation indices in fin gDNA of *piwil1* F0 crispants, within a mosaic pattern of indels, allowed studying in F0 crispants if salmon Piwil1 functions during spermatogenesis, in addition to being important for establishing and/or maintaining germ line cells. 17 of the 39 *piwil1* crispant males presented disturbances during the spermatogenic process (apparent partial loss of undifferentiated spermatogonia, type B spermatogonia and spermatocytes; delayed spermatogonial proliferation), which may explain the lower GSI of F0 crispant males during the testicular growth period in May. However, there was no obvious relation to the plasma androgen levels, which were not different between *piwil1* crispant and wt males. Jointly, these observations indicate that similar to other mammals (Vourekas et al., 2012) and teleost species (Houwing et al., 2007 and, 2008), salmon Piwil1 may have a role during spermatogenesis. Moreover, a few F0 *piwil1* crispant prepubertal testis were completely or partially devoid of germ cells. Similar to the fins of F0 *piwil1* crispant males, most likely also the juvenile testes contained spermatogonial stem cells showing either *piwil1* ^+/+^, *piwil1* ^*+/−*^ or *piwil1* ^*−/−*^. In this scenario, individual spermatogonial stem cells devoid of functional Piwil1 may have been lost at early stages of ontogenesis, resulting in GCF areas. Stem cells with two wild-type copies of *piwil1* would develop normally, and *piwil1* ^*+/−*^ stem cells may display developmental delays and/or their cellular offspring shows an elevated level of apoptosis resulting in a reduced number of spermatozoa in the tubules, but still compatible with fertility, similar to what has been reported in *piwil1* ^*+/−*^ zebrafish (Houwing et al., 2007). Considering the high level of transfer of *piwil1* mutations to the next generation, and the ability of *piwil1* ^*+/−*^ stem cell to go through spermatogenesis and produce sperm, just like *miwi* ^*+/−*^ mutant mice (Deng and Lin, 2002), further studies are required to determine if *piwil1* has a role in spermatogenesis in salmon.

We did not analyze folliculogenesis, oocyte development or other aspects of ovarian maturation in the *piwil1* F0 crispants, but ovulated females were highly mutated for *piwil1* and also eventually matured and produced eggs in a quality and quantity similar to wt females. In mammalian models, loss of PIWI proteins may not affect female germ cells, like in the mouse (Kuramochi-Miyagawa et al., 2004), or can result in sterility, as in female golden hamsters, which produce non-functional oocytes (Hasuwa et al., 2021).

Albeit highly mutated, *piwil1* crispant F0 males and females were fertile and produced offspring, allowing the generation of F1 *piwil1* KO salmon. In view of the long generation time in salmon (3 years) and in order to obtain homozygous knockout fish already in the F1 generation, we skipped the wt outcross that is commonly used when working with model species with a much shorter generation time. Instead, we produced an F1 generation by crossing highly mutated F0 crispant males and females with each other. When F1 fish were 10 months old, we assayed the gonad phenotype and genotyped the fish for *piwil1* mutations. Different indels (*in-frame* and nonsense base deletions, base insertions and substitutions) were identified, reflecting the parental mosaicism. Among them, a stop codon forming mutation (*mut4*) was also present in some fish. Analyzing the F1 gonad phenotype by histology, we could discriminate the mutations that led to loss of function (*lof* ^*-*^) of the Piwil1 (*del1*, *del5*, *del8*, *del12*, *del18*, *del20*, *ins1*, and *mut4*), while other functional mutations (*fmut* ^*+*^) did not affect Piwil1 function (*del3*, *ins9*, *mut1*, and *mut2*)*.*

All *piwil1* ^*−/−*^ (KO) testes and ovaries were germ cell free (GCF), and most *piwil1* ^*+/−*^ displayed a reduced number of germ cells, possibly reflecting a gene dose effect, similar to findings in zebrafish (Houwing et al., 2007). In zebrafish, loss of the *piwil1* ortholog *ziwi*, allows primordial germ cell (PGC) migration to the gonadal ridge and their transition into undifferentiated spermatogonia, but at the beginning of pubertal spermatogenesis, germ cells go through apoptosis and eventually die. Different from zebrafish, salmon *piwil1* ^−/−^ display GCF testes already in prepubertal juveniles, suggesting that salmon germ cells devoid of Piwil1 are lost earlier than in zebrafish. Besides, all *piwil* ^*−/−*^ F1 salmon sampled during almost 1.5 years consistently lacked germ cells and no atypical germ cell apoptosis was observed, indicating that different from zebrafish, there is no progressive germ cell loss in *piwil1* KO salmon.

The phenotype of a GCF ovary from salmon *piwil1* ^*−/−*^ F1 and *dnd* crispant females (Wargelius et al., 2016) resembles that of the rainbow trout *dnd* KO ovary (Fujihara et al., 2022): a thin and dense structure of connective tissue with rare groups of somatic cells that may represent granulosa cells (GCF *piwil1* ^*−/−*^ F1 ovaries express aromatase, *cyp19a1a*, likewise the *dnd* mutant ovaries; Wargelius et al., 2016). The resemblance in phenotype between *dnd* crispant and *piwil1* ^−/−^ females may further suggest that germ cells are lost during early ontogenesis in *piwil1* ^*−/−*^ salmon embryos, as germ cells are no longer detectable 90 days post fertilization in *dnd* KO rainbow trout (Fujihara et al., 2022). Future work will have to elucidate if the loss of germ cells in salmon occurred as early as during germ line establishment, as reported for *piwil1* KO in medaka (Li et al., 2012).

The N-domain targeted in our experiments is localized in a region conserved among vertebrates (Yuan et al., 2005; Yamaguchi et al., 2020). The loss of 4 or 6 amino in a loop structure situated between an alpha helix and a beta-sheet in the N-domain of salmon Piwil1, resulted in a non-functional Piwil1 protein. Interestingly, 3D modeling of Piwi proteins in the Gram positive bacteria, *Aquifex aeolicus* has revealed a role for the N terminal region in folding and correct functioning of the downstream PAZ domain (Yuan et al., 2005; Yamaguchi et al., 2020). Homology modeling of the observed deletion variants, *del12* and *del18,* using Swissmodel (Waterhouse et al., 2018) further suggests that proper folding might be affected. Notably, according to the predicted structure models, conformational constraints seem to cause displacement of amino acids protruding to the surface area of the protein. These deletions could therefore by steric hindrance compromise the interaction with Piwi interacting proteins in the N-terminal part of the protein such as the Tudor domain proteins, which are essential for piRNA 3’ end trimming and germplasm assembly when bound to Piwil1 (Ding et al., 2019; Yamaguchi et al., 2020), or otherwise be interfering with loading of piRNAs. Considering that in the *del3* variants only the *Val133-Met134* amino acids are affected and in the *ins9* variant*, Met134* is lost and *LAPT is* inserted between *Val133* and *Glu135,* and that these alleles are still compatible with a functional Piwil1 protein, most likely those amino acids are not essential for Piwil1 function in salmon.

In summary, we have found that knocking out *piwil1* in salmon leads to germ cell-free males and females, while partial gene loss in heterozygous (^*+/−*^) fish appears to reduce germ cell number. In addition, while we did not study a possible function of Piwil1 during salmon ovarian maturation, spermatogenesis was transiently disturbed but not blocked and the *piwil1* crispants were fertile. Finally, we found that the amino acids in the N-domain around aa129-136 seem crucial for Piwil1 function.

## Data Availability

The data presented in the study are deposited in the SRA repository, accession number: PRJNA855572.
