# Supplementary material for: The Piwil1 N domain is required for germ cell survival in Atlantic salmon
Source: Front Cell Dev Biol. 2022 Sep 19;10:977779. doi: 10.3389/fcell.2022.977779 (PMC9527287; doi:10.3389/fcell.2022.977779)
Supplement: Supplementary file 1 [file Table1.DOCX]

**Supplementary Information**

**Figure 1-** Relative expression of *vasa* in the gonads of wild type (WT), *piwil1^+/-^* (+/-) and *piwil1^+/-^* (-/-) F1 Atlantic salmon (n = 11 to 27).


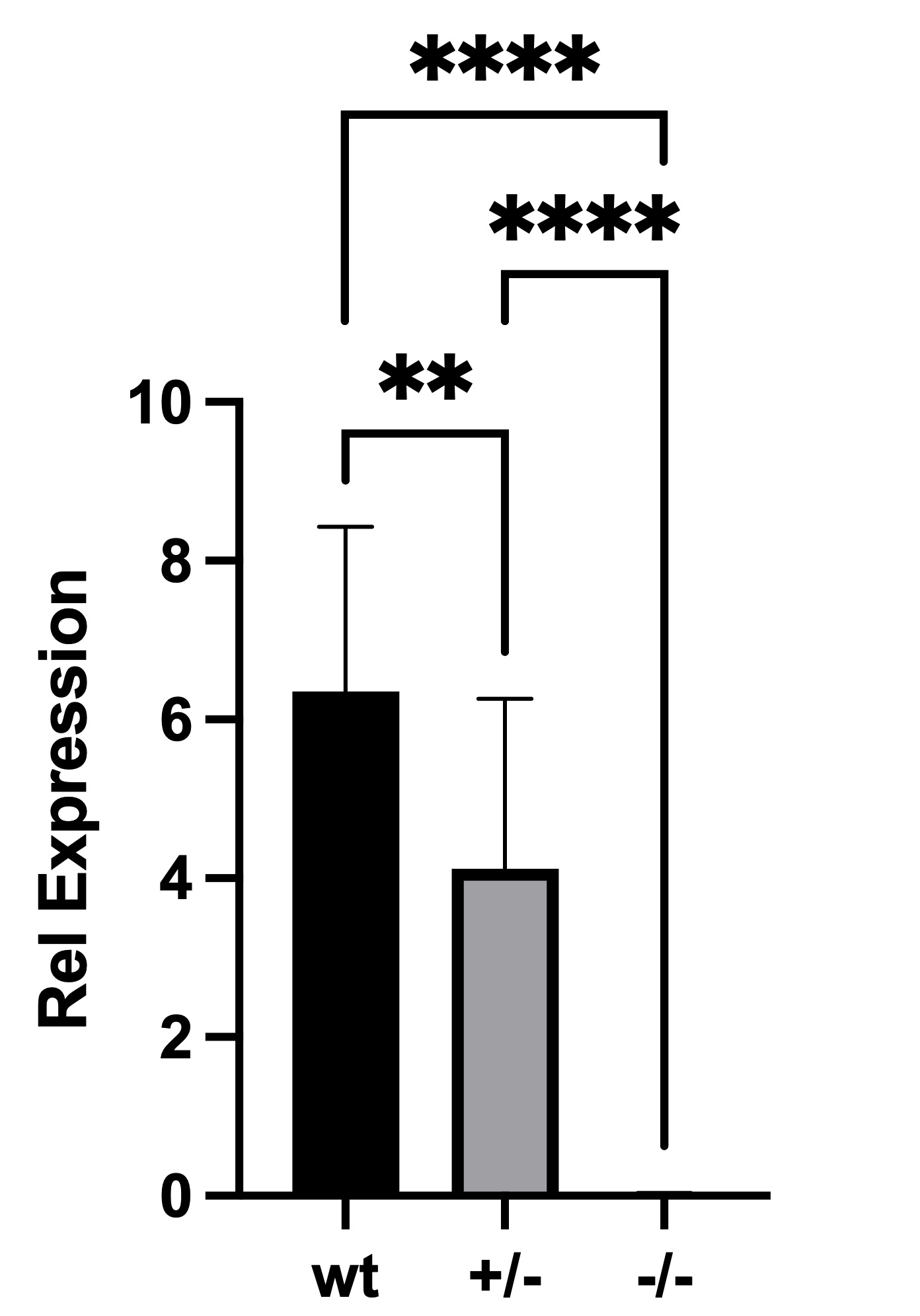


**Table 1** - Mutations rate in the *piwil1* gene of crispants (F0) revealed by miSeq deep sequencing on genomic DNA (gDNA) obtained from fin clips of mature males (M) and females (F)

| dna  # | Sex | Modified% | Frameshift % | Inframe % | Indel |  |  |  |  |  |  |  |  |
| --- | --- | --- | --- | --- | --- | --- | --- | --- | --- | --- | --- | --- | --- |
| 256 | F | 96,83 | 38.37 | 58.46 | del8>del3>del3>del6>del11>wt>del4mut2>del18>ins61mut1 | | | | | |  |  |  |
| 267 | M | 73,12 | 14.03 | 59.09 | del2>wt>del8>del3>ins1>ins9 | | |  |  |  |  |  |  |
| 275 | M | 59,79 | 22.65 | 37.13 | wt>del8>del8>ins1>del3>mut2>mut1>del5>del18>del5 | | | | | |  |  |  |
| 281 | F | 98,88 | 48.13 | 50.74 | mut2>del8>del31>mut2>del3>del2mut1>del3mut1>del1>ins3>ins1>del18>ins4mut2>del1mut1 | | | | | | | | |
| 291 | M | 70,29 | 14.93 | 55.36 | wt>del2>del8>del5>del12 | | |  |  |  |  |  |  |
| 292 | F | 96,05 | 21.63 | 74.41 | del8>del8>del32>del8>del12>del2>wt>mut2>del13>del1>del20>del5 | | | | | | |  |  |
| 300 | M | 90,98 | 51.52 | 39.45 | mut1>del8>mut2>wt>del1mut1>ins6mut1>mut2>del5>del8>del6>mut1>del5 | | | | | | | |  |
| 323 | F | 95,91 | 39.96 | 55.95 | wt>del2mut1>del12>del8>del8 | | |  |  |  |  |  |  |
| 327 | M | 80,44 | 38.80 | 41.64 | del3>del31>wt>del8>del5>del18>del3>del9 | | | | |  |  |  |  |
| 346 | M | 68,17 | 44.16 | 24.01 | del20>del18>del3>del12>del12>del7>mut1>wt>mut2>del8 | | | | | |  |  |  |
| 348 | M | 61,49 | 13.46 | 48.03 | del3>del4>del3>del28>wt>del8>del4 | | | |  |  |  |  |  |
| 353 | M | 97,40 | 61.41 | 35.99 | mut2>del18>mut5>del3>del12>wt>del8>del6>del12>mut2 | | | | | |  |  |  |
| 354 | M | 92,93 | 22.21 | 70.71 | ins6>del20>del5>del20>del7>ins1mut2>del1>del8>del10>wt>ins9>ins4mut1 | | | | | | | |  |
| 356 | M | 95,33 | 38.70 | 56.62 | del3>wt>del8>del6>del8>del31 | | |  |  |  |  |  |  |
| 359 | M | 83,73 | 32.92 | 50.80 | ins3>del31>ins9mut1>del6>del20>del3>del18>del3>wt>del8>del5>mut2>del2 | | | | | | | |  |
| 363 | M | 60,81 | 18.70 | 42.10 | del3>wt>del8>del5>del15>ins28mut5 | | | |  |  |  |  |  |
| 380 | F | 96,62 | 45.14 | 51.48 | del1>wt>del8>del3>del12>del3>del8>del1mut1 | | | | |  |  |  |  |
| 387 | F | 84,49 | 26.50 | 57.99 | mut2>ins1>ins4>del3>del8>wt>del1mut1>del3>del29 | | | | |  |  |  |  |
| 393 | F | 79,66 | 21.20 | 58.45 | del9>del5>del18>del5>del3>del8>wt>del5>del156>del9mut1 | | | | | |  |  |  |
